# Identification of diterpenoid compounds that interfere with Fli-1 DNA binding to suppress leukemogenesis

**DOI:** 10.1038/s41419-019-1363-1

**Published:** 2019-02-11

**Authors:** Tangjingjun Liu, Lei Xia, Yao Yao, Chen Yan, Yanhua Fan, Babu Gajendran, Jue Yang, You-Jun Li, Juan Chen, Jorge Filmus, David E Spaner, Eldad Zacksenhaus, Xiaojiang Hao, Yaacov Ben-David

**Affiliations:** 1 State Key Laboratory for Functions and Applications of Medicinal Plants, Guizhou Medical University, Guiyang, 550025 China; 2 The Key Laboratory of Chemistry for Natural Products of Guizhou Province and Chinese Academic of Sciences, Guiyang, Guizhou 550014 China; 3 State Key Laboratory of Phytochemistry and Plant Resources in West China, Kunming Institute of Botany, Chinese Academy of Sciences, Kunming, China; 4 Department of Anatomy, Norman Bethune College of Medicine, Jilin University, Changchun, China; 5 Biology Platform, Sunnybrook Research Institute, Toronto, Canada; 6 Department of Medicine, University of Toronto, Toronto, Ontario Canada; 7 Division of Advanced Diagnostics, Toronto General Research Institute—University Health Network, Toronto, Ontario Canada

## Abstract

The ETS transcription factor Fli-1 controls the expression of genes involved in hematopoiesis including cell proliferation, survival, and differentiation. Dysregulation of Fli-1 induces hematopoietic and solid tumors, rendering it an important target for therapeutic intervention. Through high content screens of a library of chemicals isolated from medicinal plants in China for inhibitors of a Fli-1 transcriptional reporter cells, we hereby report the identification of diterpenoid-like compounds that strongly inhibit Fli-1 transcriptional activity. These agents suppressed the growth of erythroleukemic cells by inducing apoptosis and differentiation. They also inhibited survival and proliferation of B-cell leukemic cell lines as well as primary B-cell lymphocytic leukemia (B-CLL) isolated from 7 patients. Moreover, these inhibitors blocked leukemogenesis in a mouse model of erythroleukemia, in which Fli-1 is the driver of tumor initiation. Computational docking analysis revealed that the diterpenoid-like compounds bind with high affinity to nucleotide residues in a pocket near the major groove within the DNA-binding sites of Fli-1. Functional inhibition of Fli-1 by these compounds triggered its further downregulation through miR-145, whose promoter is normally repressed by Fli-1. These results uncover the importance of Fli-1 in leukemogenesis, a Fli-1-miR145 autoregulatory loop and new anti-Fli-1 diterpenoid agents for the treatment of diverse hematological malignancies overexpressing this transcription factor.

## Introduction

Leukemogenesis involves alterations in multiple oncogenes and tumor suppressor genes as well as disruption of tumor microenvironment^1,2^. Standard therapy including surgery, chemo-, radio- and even targeted-therapy are unsuccessful in curing leukemia. Thus, more potent modalities and patient-tailored therapies are needed to eradicate malignant forms of this disease.

One major driver of leukemogenesis is the ETS transcription factor (TF), Friend leukemia integration 1 (Fli-1), originally identified as a site of common proviral integration in F-MuLV-induced erythroleukemias^3^. Activation of Fli-1 was subsequently confirmed to underlie induction of erythroleukemias by this virus^4,5^. Fli-1 was also identified as a site of specific chromosome 11;22 translocations in childhood Ewing’s sarcomas^6^. The chimeric EWS/FLI-1 fusion protein generated from this translocation is a potent oncogene^6^. Fli-1 exerts its effects by controlling the expression of genes involved in proliferation, differentiation, program cell death (apoptosis) and inflammation, all important hallmarks of cancer^7,8^. Fli-1 also promotes angiogenesis, further contributing to tumor progression^7^. Knockdown of Fli-1 in such tumors potently suppress their growth^9^ indicating that tumors driven by Fli-1 are addicted to its continuous expression. These observations point to Fli-1 as an important therapeutic target for the diverse type of malignancies driven by this oncogene^7^.

In the past decade, various methods were used to target DNA- and RNA-binding activities of EWS-Fli-1 for the treatment of Ewing Sarcomas. These efforts led to the identification of several compounds with potent anti-cancer activity^10–14^, yet none has been implemented in the clinic. There is therefore an urgent need to identify more specific and potent inhibitors of EWS-Fli-1 and/or Fli-1 with clinical utility. Toward this end, we previously performed high throughput screens to identify drugs that specifically target this TF. Several anti-Fli-1 compounds were identified and shown to block leukemic cell proliferation in culture and leukemogenesis in mouse models^10^. However, these compounds target other proteins in addition to Fli-1, and exhibited various side effects. To identify more potent and specific inhibitors, we here report on a Fli-1 inhibitor screen of a library of chemicals isolated from medicinal plants in China. We identified two chemically related diterpenoid-like compounds that suppress Fli-1 transcriptional activity and its downstream targets, leading to inhibition of B cell lymphoma in vitro and erythroleukemia in a preclinical mouse model. The inhibition of Fli-1 by these diterpenoids subsequently triggered post-transcriptional downregulation of Fli-1 protein levels through upregulation of miR-145. Thus, this work identifies novel inhibitory compounds that can be used for the treatment of cancers driven by overexpression of Fli-1.

## Results

### Identification of potent Fli-1 inhibitors from a library of compounds isolated from medicinal plants in China

**Fig. 1:**
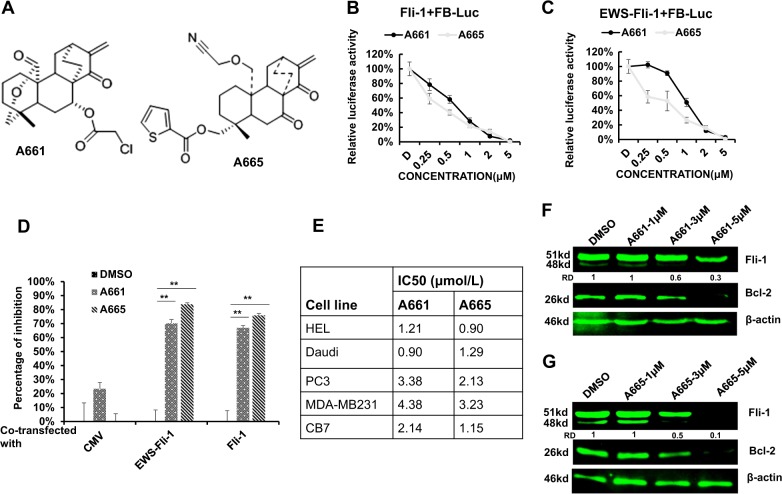
Diterpenoid compounds A661 and A665 suppress Fli-1 expression. **a** Chemical structures of the diterpenoid compounds A661 and A665. **b,** **c** A665 and A665 suppress transcriptional activity of FB-Luc, co-transfected with Fli-1 (**b**) or EWS-Fli-1 (**c**) in a dose-dependent manner. **d** A661 and A665 (2.5 µM) specifically suppress transcriptional activity of FB-Luc, but not control CMV-Luc. **e** IC50s of A661 and A665 for the indicated cell lines. **f**, **g** Western blots for Fli-1 and BCL2 in HEL cells treated with indicated doses of A661 (**f**) or A665 (**g**) for 18 h. β-actin was used as a loading control. * Denotes *P* < 0.05; **, *P* < 0.005. RD: relative density

To identify specific anti-Fli-1 compounds with low toxicity for treating tumors overexpressing this TF, we screened a library of 2000 small, highly purified compounds isolated from medicinal plants in China. As a reporter, we used a plasmid, FB-Luc, in which two Fli-1 binding sites were placed upstream of a minimum promoter of the luciferase PGL-4.28 plasmid^10^. HEK293T cells stably expressing Fli-1 and FB-Luc plasmids were established and used for the screen. Several compounds were identified. Among these, A661 and A665 (Fig. 1a), are structurally related to a family of natural diterpenoids^15^. These compounds strongly inhibited luciferase activity in HEK293T cells co-transfected with FB-Luc and MigR1-Fli-1 relative to control MigR1 expression vector in a dose-dependent manner (Fig. 1b, c). The compounds also inhibited luciferase activity following co-transfection of FB-Luc with MigR1-EWS-Fli-1. Suppression was Fli-1 specific; it was low or marginal with a control CMV-Luc reporter plasmid lacking Fli-1 binding sites (Fig. 1d).

### Inhibition of Fli-1 by A661 and A665 in erythroleukemic cells induces apoptosis, cell cycle arrest, and erythroid differentiation

A661 and A665 inhibited growth of cell lines derived from solid tumors such as breast (MDA-MB-231) and prostate (PC3) as well as from leukemic lines (CB7, Daudi, HEL) with IC50 ranging from 0.9-4.3 μM (Fig. 1e). Among these leukemic cell lines, HEL, Daudi, and CB7 express Fli-1 while the rest are either negative or produce negligible levels of this TF^16^. SiRNA-mediated knockdown of Fli-1 in both HEL and CB7 cells was previously shown to inhibit cell proliferation through induction of apoptosis and erythroid differentiation^9^. Inhibition of proliferation by A661/A665 in Fli-1-negative cells suggests that these compounds target additional proteins required for cell proliferation (see below). However, in tumors that are driven by Fli-1, this oncogene is the major target of these compounds.

Treatment with A661 or A665 downregulated Fli-1 protein expression in HEL cells (Fig. 1f, g) in a dose-dependent manner. Fli-1 is thought to autoregulate itself through several ETS binding sites in its promoter^17,18^. In accordance, both compounds significantly increased mRNA levels of *fli-1* (Supplemental Fig. 1).

Compounds A661 and A665 induced rapid apoptotic cell death in erythroleukemic HEL and CB7 cells, 24 h post-treatment (Supplemental Fig. 2A). Consistent with this, inhibition of Fli-1 resulted in reduced expression of Bcl-2, a known downstream target of this TF (Fig. 1f, g). In addition to apoptosis, A661 and A665 induced cell growth inhibition, which was associated with a significant increase in the percentage of cells in G2 (Supplemental Fig. 2B). There was also some effect on S phase that was variable between cell lines (Supplemental Fig. 2B). Indeed, A661 and A665 induced cyclin-dependent kinase inhibitor p27^kip1^ protein expression involved in erythroid differentiation, whereas the Fli-1 agonist TPA suppressed p27^kip1^ expression (Supplemental figure 3A)^16^.

We previously showed that Fli-1 knockdown induces erythroid differentiation^9^. To determine whether A661 and A665 affect the same differentiation program as Fli-1 knockdown, we quantified the expression of a subset of its target genes that promote erythroid maturation^10^. Specifically, Fli-1 negatively regulates transcription of GATA1 and SHIP-1 involved in erythroid differentiation, and positively regulates transcription of glycoprotein 6 (GP6) required for megakaryocyte development^17–22^. Consistent with this, mRNA levels of the erythroid genes GATA1 and SHIP-1 was induced by both A661 and A665 (Supplemental Fig. 3B, C), whereas expression of GP6 was suppressed (Supplemental Fig. 3D). Expression of β-globin and glycoporin A (GYPA), both markers of erythroid differentiation, was induced by both compounds, but more by A665 (Supplemental Fig. 3E, F). While MAPK/ERK phosphorylation was slightly affected by A661 and A665, expression of c-MYC, an important regulator of erythroid differentiation^23,24^, was significantly downregulated (Supplemental Fig. 4A, B). Overall, these results demonstrate that pharmacological inhibition of Fli-1 by A661 or A665 closely mimics the effect of its genetic ablation by siRNA, indicating that these compounds exert their cellular and molecular effects to a large degree through inhibition of Fli-1.

### Computational analysis predicts that A661 and A665 bind near the DNA-binding sites of Fli-1, leading to transcriptional suppression

**Fig. 2:**
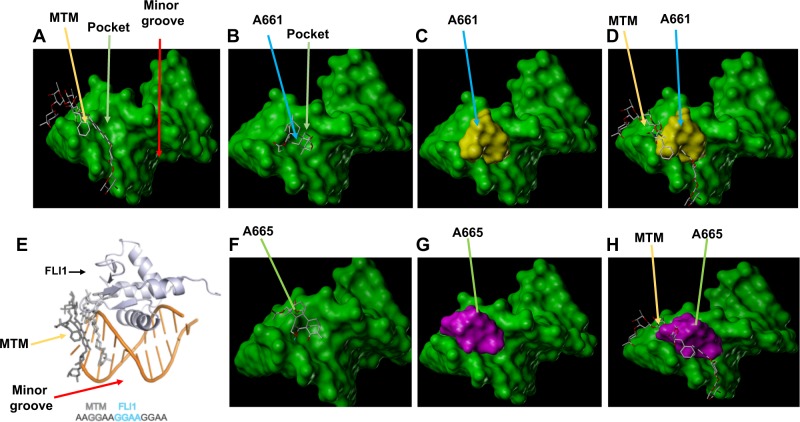
A661 and A665 are predicted to bind a pocket domain near the minor groove within the Fli-1 DNA-binding sites. **a** Binding of mithramycin (MTM) near the minor pocket of EWS-FLI-1 binding site. Relative binding of MTM and A661 (**b**–**d**), MTM and A665 (**f**–**h**) to FLI-1 binding site. **e** Relative binding of MTM and FLI-1 to DNA within the Fli-1 binding site

**Fig. 3:**
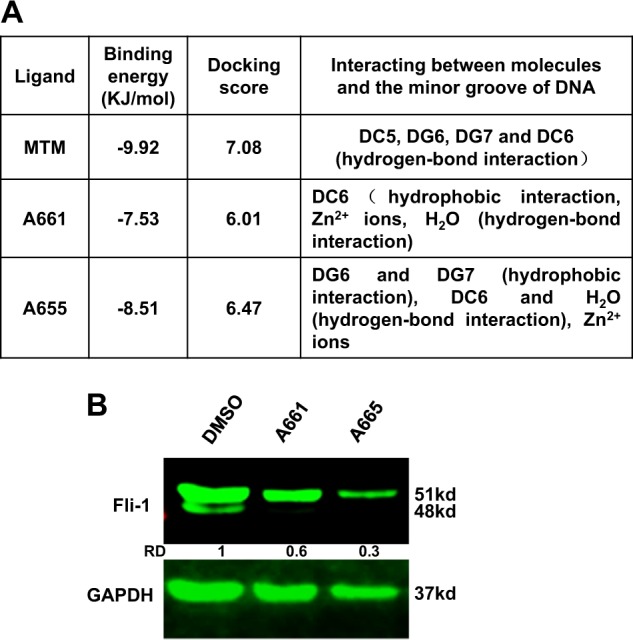
High affinity of A661 and A665 compounds binding to the Fli-1 consensus DNA-binding sites. **a** Properties of A661 and A665 binding affinities to the Fli-1 binding sites. **b** Western blot of HEL cells treated with 2.5 μM of A661 or A665. GAPDH was used as loading control

Hou et al.^25^ have recently demonstrated strong affinity of mithramycin (MTM), a known inhibitor of EWS-Fli-1, to a GGAA repeat near the minor groove of DNA, which is recognized by this chimeric transcription factor (Fig. 2a, e). This interaction subsequently perturbed binding of the ets domain of EWS-FLI1 to the major groove of this DNA recognition site (Fig. 2e). Through docking analysis, we found that both A661 (Fig. 2b–d) and A665 (Fig. 2f–h) bind within a pocket near minor groove in vicinity of the MTM binding site. This comparative analysis revealed that A661 and A665 have similar binding affinity and docking score with MTM (Fig. 3a). From this analysis, A665 is expected to be a much better inhibitor of Fli-1 than A661; A665 has a predicted binding energy of -8.51KJ/Mol compared to A661 with -7.53KJ/Mol (Fig. 3a). Consistent with this analysis, western blot analysis revealed that Fli-1 expression was more downregulated by A665 than by A661 (Fig. 3b). MTM showed hydrogen-bond interactions with 4 nucleotides: DC5, DG6, DG7 and DC6. A661 in addition to hydrophobic interactions with nucleotide DC6 also formed hydrogen-bond interaction with H_2_O and coordination bonds with Zn^2+^ ions (Fig. 3a). A665 formed hydrophobic interaction with nucleotides DG6 and DG7, hydrogen-bond interaction with H_2_O and coordination bonds with Zn^2+^ ions. Thus, A661/A665 may represent novel anti-Fli-1 compounds with unique interaction with the DNA-binding sites of Fli-1.

### Post-transcriptional regulation of Fli-1 by miR-145

**Fig. 4:**
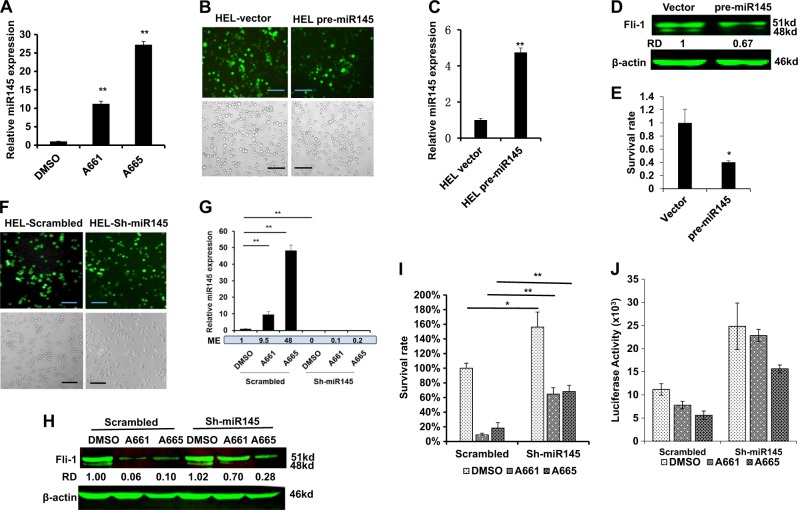
Fli-1 inhibiting compounds induce miR145 in leukemic cells. **a** Q-RT-PCR analysis of miR145 expression in HEL cells 12 h post treatment with A661 or A665 (5 µM). **b** Images of HEL cells transduced with pre-miR145 or control lentivirus vector after sorting for GFP positive cells and subsequent selection for two days in puromycin (1 μg/ml). Upper panels show GFP expression; lower panel, bright field view of the cells (original magnification X20, scale bars,100 μm). **c**, **d** Q-RT-PCR (**c**) and western blot (**d**) analysis of miR145 expression in transduced pre-miR145 and control HEL cells. **e** Survival of HEL cells determined by MTT assay 72 h post transduction with miR145 versus control lentiviruses. Representative results from 3 experiments, each in triplicates (n = 3). **f** Photographs of HEL cells transduced with sh-miR145 or control scrambled lentiviruses. Upper panels show GFP expression; lower panel macroscopic view of the cells (magnification ×200). **g** Q-RT-PCR analysis of miR145 expression in sh-miR145-5p sponge or scrambled transduced HEL cells after treatment for 12 h with A661 or A665 (5 µM). ME: Mean expression. **h** Western blot for expression of Fli-1 in HEL cells transduced with sh-miR145-5p sponge or scrambled lentiviruses, 12 h post treatment with A661 or A665 (5 µM). **i** Survival of HEL cells determined by MTT assay 48 h after transduction with sh-miR145-5p or control DNA, treated with the indicated compounds (5 µM). * Denotes *P* < 0.05; **, *P* < 0.005. **j** Higher Fli-1 activity detected in sh-miR145-HEL cells than in control scrambled-HEL cells after transfection with FB-Luc plasmid and treatment with the indicated compounds (5 μM)

The observed downregulation of Fli-1 protein expression by A661 and A665 (Fig. 1f, g) was not at the transcriptional level (Supplemental Fig. 1). The proteasome inhibitor MG132 did not rescue Fli-1 expression indicating that its reduced expression was not due to protein degradation (Supplemental Fig. 5). Previous studies demonstrated that microRNA-145 (miR-145) controls Fli-1 protein translation^26–29^. Remarkably, we found that treatment with A661 and A665 resulted in a significant upregulation of miR145 expression, with A665 having a stronger effect (Fig. 4a). To validate the role of miR145 in drug-induced inhibition of Fli-1, we transduced HEL cells with a pre-miR145 lentivirus or control vectors, sorted GFP-positive cells by flow cytometry and photographed the cells two days post selection with puromycin (Fig. 4b). Significantly lower viability was seen with the pre-miR-145 transduced cells compared to vector control (Fig. 4b). Furthermore, miR145 overexpression (Fig. 4c) significantly suppressed Fli-1 protein level (Fig. 4d) and reduced cell survival (Fig. 4b, e).

To complement these results, we knocked down endogenous miR145 by transducing GFP tagged lenti-sh-miR145 or scrambled control vector into HEL cells. GFP positive sh-miR145 and scrambled control expressing cells exhibited similar cell number (Fig. 4f). Expression of miR-145, which was induced by A661 or A665, was completely diminished by sh-miR145 expression (Fig. 4g). Moreover, expression of the Fli-1 protein, which was suppressed by A661 or A665, was rescued by sh-miR145 (Fig. 4h). In accordance, treatment of sh-miR145 expressing cells with A661 or A665 resulted in a higher survival rate (Fig. 4i) and increased Fli-1 reporter activity following transient transfection with the FB-Luc gene (Fig. 4j). Moreover, miR-145 expression was not induced in Fli-1 negative K562 cells (Supplemental Fig. 6), demonstrating that A661 and A665 induce miR145 via Fli-1, and thus suppress the growth of leukemic cells at least in part through a Fli-1-miR145 auto-inhibition loop (see below).

### Fli-1 negatively regulates miR-145 transcription

**Fig. 5:**
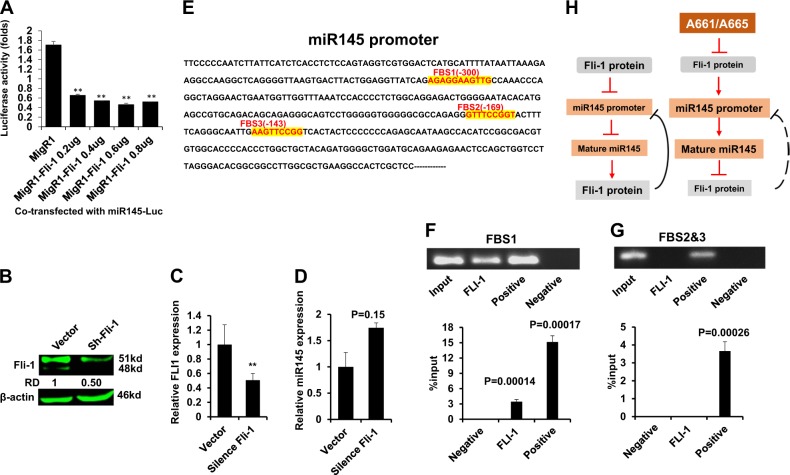
Fli-1 negatively regulates the miR-145 promoter. **a** Promoter activity was determined in HEK293T cells transfected with miR145-Luc (0.2 µg) and indicated plasmids. (**b**–**d**) Immunoblotting for Fli-1 (**b**) mRNA (**c**) or (**d**) miR145 following transduction of the sh-Fli-1 lentivirus. **e** Promoter sequence of FLI1 with the FLI1 binding sites (FBS1-3) highlighted in red. **e**, **f** Chromatin immunoprecipitation (ChIP) analysis for the regions containing FBS1 (**f**) and FBS2-3 (**g**). **h** Depicted model of A661/A665. In leukemic cells, high Fli-1 suppresses of the miR-145 promoter, leading to higher Fli-1 expression. The diterpenoid compounds A661 and A665 suppress Fli-1 expression either directly or through an upstream factor. Fli-1 downregulation in turn depresses the *miR-145* promoter, thereby leading to miR145-mediated inhibition of Fli-1 translation

The aforementioned results suggested that Fli-1 negatively regulates the miR-145 promoter as had previously been reported for EWS-Fli-1^29^. We tested this possibility using a miR-145 promoter reporter plasmid, miR145-Luc. Overexpression of Fli-1 significantly suppressed the miR-145 promoter in this assay (Fig. 5a). Conversely, knockdown of Fli-1 via shRNA significantly increased endogenous expression of miR-145 (Fig. 5b–d).

The human miR145 promoter contains 3 ETS-binding sites (FBS1-FBS3; Fig. 5e). Chromatin immunoprecipitation (ChIP) analysis confirmed binding of FLI1 to a region surrounding FBS1, but not FBS2-3 (Fig. 5f, g). As a positive control, antibody for RNA polymerase II immunoprecipitated a fragment of the miR-145 promoter containing all three sites. Together, these results suggest a model in which in leukemic cells, high Fli-1 expression suppresses miR145 transcription, leading to enhanced Fli-1 translation that further suppresses miR145 (Fig. 5h left). Upon drug treatment, Fli-1 inhibition induces miR-145 that creates the opposite effect, leading to a negative feedback loop that extinguishes Fli-1 expression (Fig. 5h right).

### Fli-1 inhibition via A661/A665 or shRNA suppresses growth of leukemic cells in culture and in a preclinical model of leukemia

**Fig. 6:**
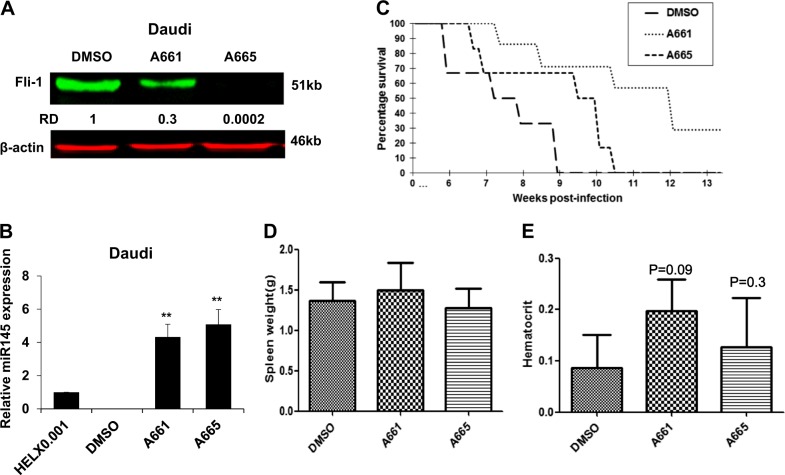
Fli-1 inhibiting compounds suppress leukemic cell growth in vitro and in vivo. **a**, **b** Fli-1 knockdown via shRNA in Daudi cells (**a**) resulted in significant upregulation of miR145 expression (**b**). **c** Both A661 and A665 inhibit leukemogenesis in a mouse model of leukemia in which Fli-1 is induced by retroviral insertional mutagenesis. Newborn BALB/c mice (*n* = 7 per group) were infected with F-MuLV and 6 weeks later treated with A661 or A665 (3 mg/kg) every other day for a total of six injections. Latency to death was used to plot a Kaplan-Meier survival curve (**d**, **e**). Spleen weights (**d**) and hematocrit values (**e**) of leukemic mice at time of scarification

**Fig. 7:**
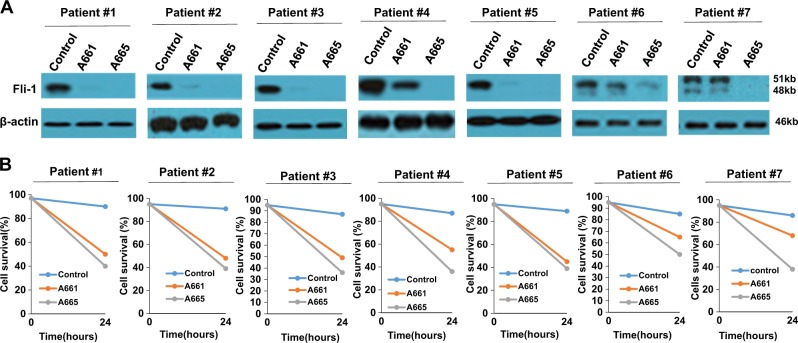
Fli-1 inhibiting compounds efficiently suppress Fli-1 expression and kill B-CLL cells. **a** Western blot for Fli-1 expression in B-CLL cells isolated from patients 1–7, 18 h post A661 or A665 (5 µM) treatment. **b** Viability of B-CLL cells after treatment with A665 or A661, using Trypan blue dye exclusion assays

Fli-1 expression is elevated in B-cell and myeloid tumors^7,30^. Both A661 and A665 significantly reduced Fli-1 protein expression in the B-cell lymphoma cells line Daudi (Fig. 6a). Fli-1 inhibition by these drugs was associated with a dramatic upregulation of miR-145 (Fig. 6b) and reduced survival in culture (Fig. 1e). A drastic decrease in Fli-1 expression was also detected in B-chronic lymphocytic leukemia (B-CLL) cells isolated from 7 independent patients, 12 h post incubation with A661 or A665 (Fig. 7a). Twenty-four-hour treatment with A661/A665 led to a significant reduction in cell viability (Fig. 7b), demonstrating the utility of these compounds for the treatment of B-cell lymphoma.

To investigate the ability of A661 and A661 to inhibit leukemogenesis in vivo, we used an animal model of Friend-Murine leukemia virus (F-MuLV)-induced erythroleukemia, driven by Fli-1^10^. In this model, F-MuLV injection into newborn Balb/c mice induces transformation 4–6 weeks post-infection^31^. Mice were then treated every other day with A661 or A665 (3 mg/kg) for a period of 12 days. Treatment of leukemic mice (*n* = 7) with A661 or to a lesser extent with A665 for this short period resulted in significant inhibition of leukemogenesis^10,16^. While the size of spleens was similar in control versus drug-treated cells, mice treated with A661/A665 were less anemic, an indication of tumor inhibition (Fig. 6d, e). Thus, A661 and A665 exhibit anti-leukemic activity against leukemias driven by Fli-1 overexpression.

## Discussion

Fli-1 is overexpressed in various cancers and diseases rendering it an important target for drug development^7^. By screening a library of compounds isolated from medicinal plants in China, we now identified two structurally related diterpenoids, A661 and A665, with potent anti-Fli-1 activity. These inhibitors downregulate Fli-1 protein and further extinguish its expression post-transcriptionally as miR-145, a suppressor of Fli-1 protein translation, is upregulated upon drug-mediated inhibition of Fli-1. A661 and A665 induced differentiation and apoptosis and attenuated the growth of B-cell lymphomas expressing high levels of Fli-1 in vitro, and erythroleukemia in a preclinical model in vivo. Thus, these drugs exert very similar effects as shRNA-mediated knockdown of Fli-1. The ability of A661 and A665 to block growth of B-cell lymphomas from multiple independent patients as well as leukemogenesis in a preclinical model point to their potential application as drugs for the treatment of leukemias and other cancers driven by Fli-1 overexpression.

The anti-Fli-1 diterpenoids displayed high affinity to the DNA-binding sites of FLI1, a region that was previously reported to be targeted by Mithramycin, a potent inhibitor of the EWS-FLI1 fusion oncoprotein^25^. This binding inhibits Fli-1 function leading to transcriptional modulation of downstream target genes involved in cell growth, differentiation, and apoptosis. Among these targets is miR-145, which was previously reported to be regulated by EWS-Fli-1^26-29^. MiR-145 targets Fli-1 as well as other genes that play critical roles in progression of various cancers including those of the prostate, breast and colon^32–35^. Our study demonstrates that Fli-1 inhibition by the diterpenoid compounds upregulates miR-145 expression, leading to downregulation of Fli-1 and consequently to inhibition of leukemia. High miR145 expression induced by low Fli-1 likely further suppresses growth by targeting other genes such as the RAS pathway, TGFBR2 and others^32–37^. ShRNA-mediated knockdown of miR-145 in drug-treated leukemias only partially rescued survival, indicating the importance of other Fli-1 targets during leukemogenesis. Thus, Fli-1 inhibition blocks tumor growth in part by upregulating miR145, which is kept in check in untreated tumors by high levels of Fli-1. How do A661 and A665 inhibit Fli-1? The simplest model is that these compounds, like mithramycin^25^, directly interact with the Fli-1 DNA-binding sites to inhibit its function. However, we cannot rule out the possibility that A661 and A665 inhibit Fli-1 indirectly either by blocking an upstream regulator. Future analysis is required to confirm a direct binding of A661 and A665 to Fli-1 or identify new upstream targets. However, we showed that these drugs in part set in motion a Fli-1–miR-145 autoregulatory loop that extinguishes Fli-1 expression and suppresses leukemia.

Our study confirms the negative regulation of the miR-145 promoter by Fli-1, and further shows that it is mediated by the FBS1 element in the miR145 promoter. We show that inhibition of Fli-1 by A661/A665 upregulated Fli-1 transcription. This result suggests that Fli-1 may negatively regulate itself, likely through ETS binding sites in its promoter^17,18^.

We demonstrated that A661 and A665 inhibit proliferation not only of cancer cells expressing Fli-1, but also of cancer cells lacking Fli-1 expression. This anti-proliferative activity is mediated through other target(s) in this context. Indeed, a similar diterpenoid derivative, 15-oxospiramilactone, was previously reported to block the WNT signaling in breast cancer cells^15^, and this finding has been confirmed in our study (unpublished data). Our compounds in part by suppressing Fli-1 and  the WNT pathways may cooperatively exert better inhibitory response than each target alone. Thus, A661 and A665 may offer an excellent new treatment for tumors, such as triple-negative breast cancer^37^, in which both Fli-1 and MYC drive cell survival and proliferation.

In summary, two anti-Fli-1 diterpenoid compounds identified in this study, A661 and A665, induce cell cycle arrest and apoptosis of leukemic cells in culture and inhibit leukemogenesis in vivo. These inhibitors disrupt an autoregulatory loop that maintains high level of Fli-1 via Fli-1-induced suppression of its negative regulator, miR145. These diterpenoids may provide important new tools for the treatment of leukemia and lymphomas driven by overexpression of Fli-1.

## Materials and methods

### Cell lines

Mycoplasma negative cell lines originated from erythroleukemia (CB7 and HEL), human embryonic kidney (HEK293T), B-cell lymphoma (Daudi) were maintained in Dulbecco’s Modified Eagle Medium supplemented with 5% fetal bovine serum (HyClone, GE Healthcare, Australia). Human B-CLL cells were isolated and grown, as described previously^38^.

### Leukemia induction and in vivo compound treatment

Newborn BALB/c mice were inoculated intraperitoneally (i.p.) by F-MuLV as described^16^. Six weeks post viral infection, mice were injected i.p., every other day for a total of six inoculations with A661 or A665 compounds (3 mg/kg body weight) or control DMSO and monitored for development of severe leukemia. Mice showing signs of late-stage disease were sacrificed and % survival was calculated as described^16^.

### Cell cycle and apoptosis

Apoptosis and cell cycle analysis were described elsewhere^16^. In brief, cells were incubated with compounds or DMSO for 48 h and cells then washed by cold PBS. For apoptosis experiment, cells were stained by Annexin V and PI apoptosis detection Kit (BD Biosciences, Franklin Lakes, NJ) following the kit guidelines and analyzed by flow cytometer. For cell cycle analysis, cells were fixed by cold 75% ethanol overnight at −20 °C, washed by cold PBS, stained in PI for 40 min at 37 °C, then analyzed by flow cytometer.

### Compound screening, luciferase assay, and promoter analysis

A collection of 2000 compounds, isolated from Chinese medicinal plants, was used to screen for anti-Fli-1 activity, as described^16^. In brief, HEK293T cells were co-transfected with FB-Luc (1.25 μg) and MigR1 (1.25 μg) or MigR1-Fli-1 (1.25 μg) expression vector for 24 h using Lipofectamine 2000 (Life Technology, Beijing, China) following the manufacturer’s protocol. In these transfection experiments, renilla luciferase was used as internal control to determine transfection efficiency, according to manufacturer recommendations (Promega, Fitchburg, USA). The transfected cells were plated into 96 well plates, incubated for 12 h and then treated with compounds (5 μM) or other indicated compounds for additional 12 h. Luciferase activity was determined, as described^9^. MiR145 promoter fused to luciferase gene (miR145-Luc) was obtained from Kenneth Kosik (addgene, Cambridge, MA, USA, plasmid # 21500)^39^.

### Drug studies

Triplicates of cancer cell lines (1 × 10^4^) were plated in 96 well plates and treated with various concentration of compounds for 3 days. Cells were then subjected to Tetrazolium dye (MTT) assay, as described^16^. The IC50 (the concentration of compound required to reduce 50% of cell viability) was calculated based on reading between drug and DMSO treated cells.

### Western blotting, immunoprecipitation, and inhibitory compounds

Methods for Western blotting was described elsewhere^16^. Polyclonal rabbit antibody for Fli-1 were obtained from Abcam (Abcam, Cambridge, UK); ERK and phospho-ERK from Cell Signalling Technology (CST, Danvers, MA01923), β-actin, β-Tubulin, BCL-2 from Proto-Technology (Protein-Tech, Bucuresti, Romania) and goat-anti-mouse, and goat anti-rabbit HRP-conjugated (Promega). Antibody dilution was according to the manufacturer instructions. Imaging of proteins was performed using Oddessy system (Li-Cor biosciences, Lincoln, USA). In experiments described in Fig. 7a, we used ECL detection system for western blotting, as described^9^.

### Total RNA and microRNA isolation and quantitative real-time PCR

Total RNA was isolated from cell lines with TRIzol (Invitrogen Life Technologies, Carlsbad, USA) according to the manufacturer’s protocol. A NanoDrop 2000 spectrophotometer (Thermo Scientific, USA) was used to measure RNA concentration. Reverse transcription reactions were performed with PrimeScript RT Reagent kit (Takara, Dalian, China), and quantitative real-time PCR (qRT-PCR) was performed using FastStart Universal SYBR Green Master (Roche, Mannheim, Germany) and the Step One Plus Real-time PCR system (Applied Biosystems, Singapore, Singapore). Expression was normalized to β-actin level. Primer sequences were as followed in Supplemental Table I. For miR-145 detection, miRNA isolated from cell lines using miRNA extract kit (Haigene, Haerbing, China), according to the manufacturer’s protocol. Reverse transcription reactions were performed with RevertAid First Strand cDNA Synthesis Kit (Thermo scientific, Lithuania, EU). miRNA-specific primers were purchased from RiboBio (RiboBio, Guangzhou, China), and the relative miR-145 expression level was normalized to U6. The 2-ΔΔCt method was used for relative quantification. All experiments were performed in biological triplicates, each in triplicates (*n* = 3).

### Plasmid construction, lentiviral production, and infection

The pre-miR-145 expression construct was generated by cloning pre-miR-145 sequence (Supplemental Table II) into the AsiSI-MluI sites of pLent-puro-GFP (Vigenebio, Shandong, China). A Fli-1 shRNA expression construct was generated by cloning Fli-1 4in1shRNA sequence (Supplemental Table II) into the BbsI-BcuI sites of the PHS-4in1shRNA-GFP lentivirus vector (Vigenebio). For lentivirus production, packaging plasmids psPAX2 and PMD2.D (a gift from Didier Tronon, Addgene plasmid # 12259 and 12260) and pLent-puro-GFP-mir145 or Fli-1 PSH-4in1shRNA-GFP were co-transfected into HEK293T cells, as described^16^. 48 h post transfection, supernatants were harvested, viruses were centrifuged at 2500 × *g* for 10 min and filtered through 0.45 µm filters. For infection, HEL cells were cultured and grown in the presence of fresh supernatant of lentivirus producing cells. After 24 h infection, medium was changed, and cells either sorted for GFP positive cells and/or were grown in presence of puromycin (Solanbio, Beijing, China), until -resistant cells were obtained.

The Sh-miR145 expression construct (Has-miR-145-5p) was generated by cloning Sh-miR145 sequence (Supplemental Table I) into the SgfI-MluI sites of PAV-CAG-MIR plasmid (Vigenebio). Has-miR-145-5p sponge or PAV-CAG-MIR vector was transfected into HEL cells with ViaFect Transfection Reagent (Promega, Madison, USA), following the manufacturer’s protocol. GFP- positive cells were sorted using fluorescence-activated cell sorting (FACS) 2 days post-transduction. After 10 days in culture, cells were sorted again for selection of high GFP expressing cells.

### Chromatin immunoprecipitation (ChIP) and quantitative PCR

HEL cells (1 × 10^6^) were washed and crosslinked as previously described^10^. Fixed cells were washed and resuspended in lysis buffer Magna Chip G kits (Millipore, MA, USA) and sonicated using the sonics vibra VCX150 (Scientz biotechnology, Ningbo, China). A fragmented chromatin aliquot was removed for input control. Protein G sepharose beads were added and incubated for 1 h. Immunoprecipitations were performed overnight at 4 °C with 1 μg of Fli-1 (Abcam), 1 μg of anti-RNA Polymerase II (Millipore/MERK, Darmstadt, Germany) or nonspecific normal rabbit immunoglobulin G (IgG; Abcam) antibodies. Precipitates were washed, and reverse crosslinking followed instruction for Magna Chip G kits (Millipore). DNA was incubated with proteinase K at 50 °C for 2 h, purified with phenol chloroform and resuspended in TE buffer. Real-time PCR was performed to amplify two *mir145* promoter regions containing the Fli-1 binding site 1 FBS1(position −482 to −205, primers in Supplemental Table II) and FBS2/FBS-3 (position −231 to +19). The percentage of input was calculated by Q-RT-PCR based on the intensity of amplified *fli-1* DNA divided by the amplified input DNA.

### Animal survival experiments and statistical analysis

Mice survival rates were computed and plotted according to the nonparametric Kaplan–Meier analysis. Statistical analysis was performed using the two-tailed Student *t*-test with significance considered at *P* < 0.05, and by analysis of variance using Origin 3.5 software (Microcal Software, Northampton, MA, USA).

### Computation docking analysis

The 3-dimensional structures of A661 and A665 were analyzed and drawn in chen sketch. The protein Crystallographic structure of FLI-1 (PDB ID: 5JVT) was obtained from www.rcsb.org. Auto Dock tools 1.5.6. were used to compute molecular docking simulations, as described^40^.

### Animal care

Animal care was in accordance with guidelines of the Guizhou Medical University and China Council of Animal Care.

## Supplementary information

Supplemental Figures
