# Supplementary material for: Identification of diterpenoid compounds that interfere with Fli-1 DNA binding to suppress leukemogenesis
Source: Cell Death Dis. 2019 Feb 11;10(2):117. doi: 10.1038/s41419-019-1363-1 (PMC6370842; doi:10.1038/s41419-019-1363-1)
Supplement: Supplementary file 1 — Supplemental Figures [file 41419_2019_1363_MOESM1_ESM.pptx]

## Slide 1
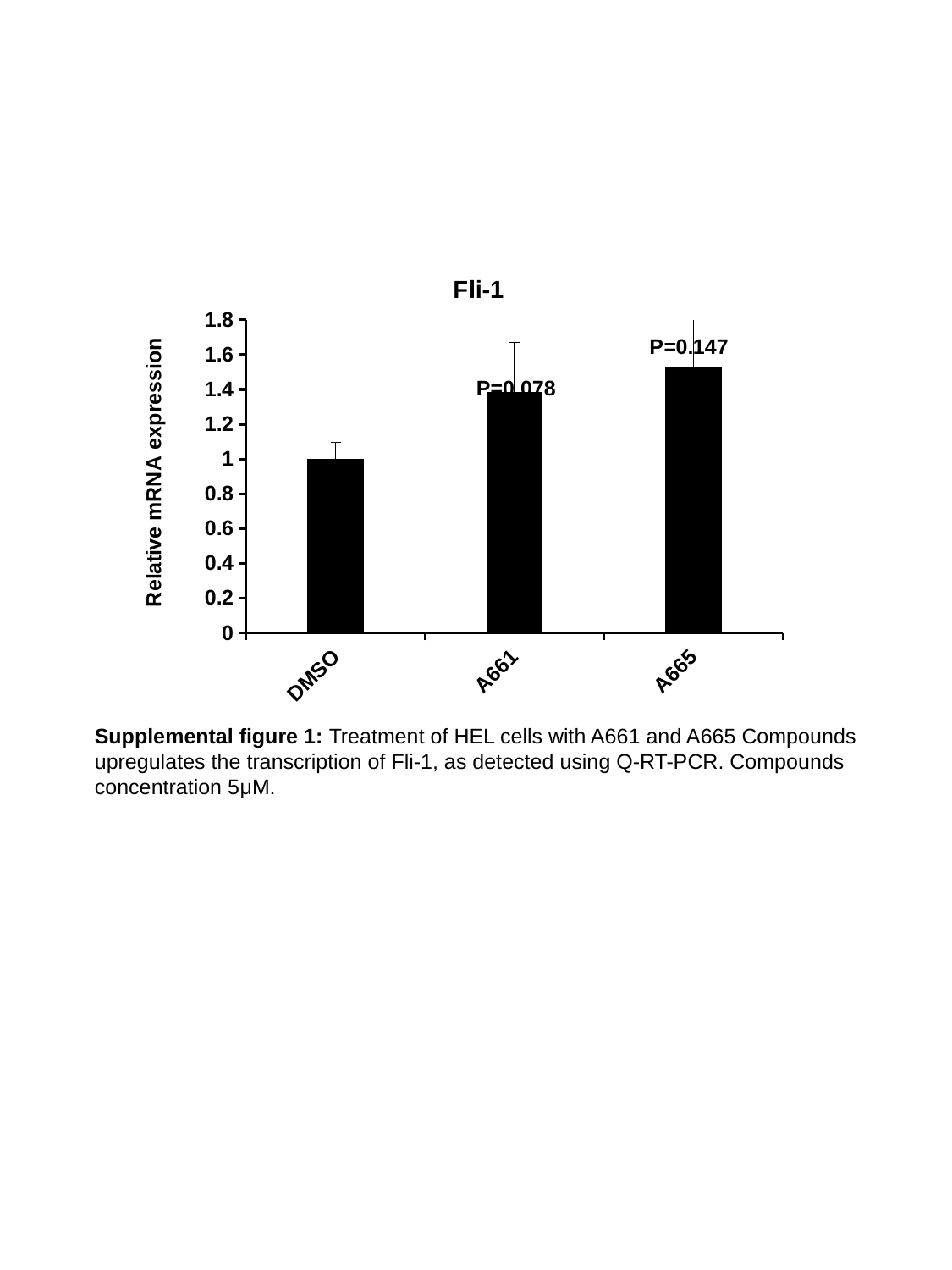

### Chart: Fli-1
| Category | RQ |
|---|---|
| DMSO | 1.0 |
| A661 | 1.3866963956210097 |
| A665 | 1.533081978526959 |P=0.078
Supplemental figure 1: Treatment of HEL cells with A661 and A665 Compounds upregulates the transcription of Fli-1, as detected using Q-RT-PCR. Compounds concentration 5μM.

## Slide 2
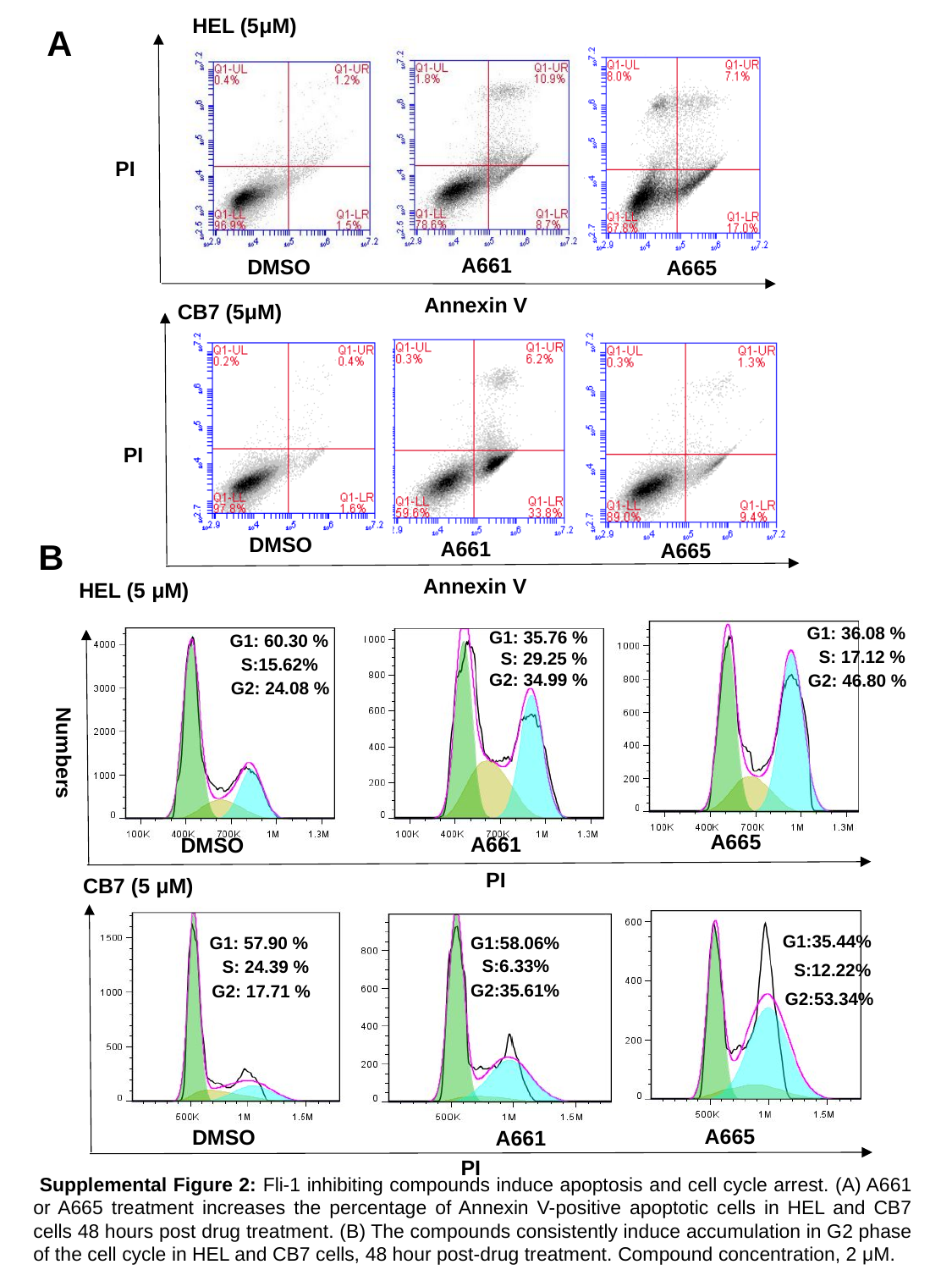

HEL (5μM)
PI
A661
DMSO
Annexin V
A665
A
 CB7 (5μM)
PI
DMSO
A661
Annexin V
A665
B
HEL (5 μM)
 G1: 60.30 %
 S:15.62%
 G2: 24.08 %
DMSO
 G1: 36.08 %
 S: 17.12 %
 G2: 46.80 %
A665
 G1: 35.76 %
 S: 29.25 %
 G2: 34.99 %
Numbers
A661
PI
CB7 (5 μM)
 G1:35.44%
 S:12.22%
 G2:53.34%
A665
 G1: 57.90 %
 S: 24.39 %
 G2: 17.71 %
 G1:58.06%
 S:6.33%
 G2:35.61%
DMSO
A661
PI
 Supplemental Figure 2: Fli-1 inhibiting compounds induce apoptosis and cell cycle arrest. (A) A661 or A665 treatment increases the percentage of Annexin V-positive apoptotic cells in HEL and CB7 cells 48 hours post drug treatment. (B) The compounds consistently induce accumulation in G2 phase of the cell cycle in HEL and CB7 cells, 48 hour post-drug treatment. Compound concentration, 2 μM.

## Slide 3
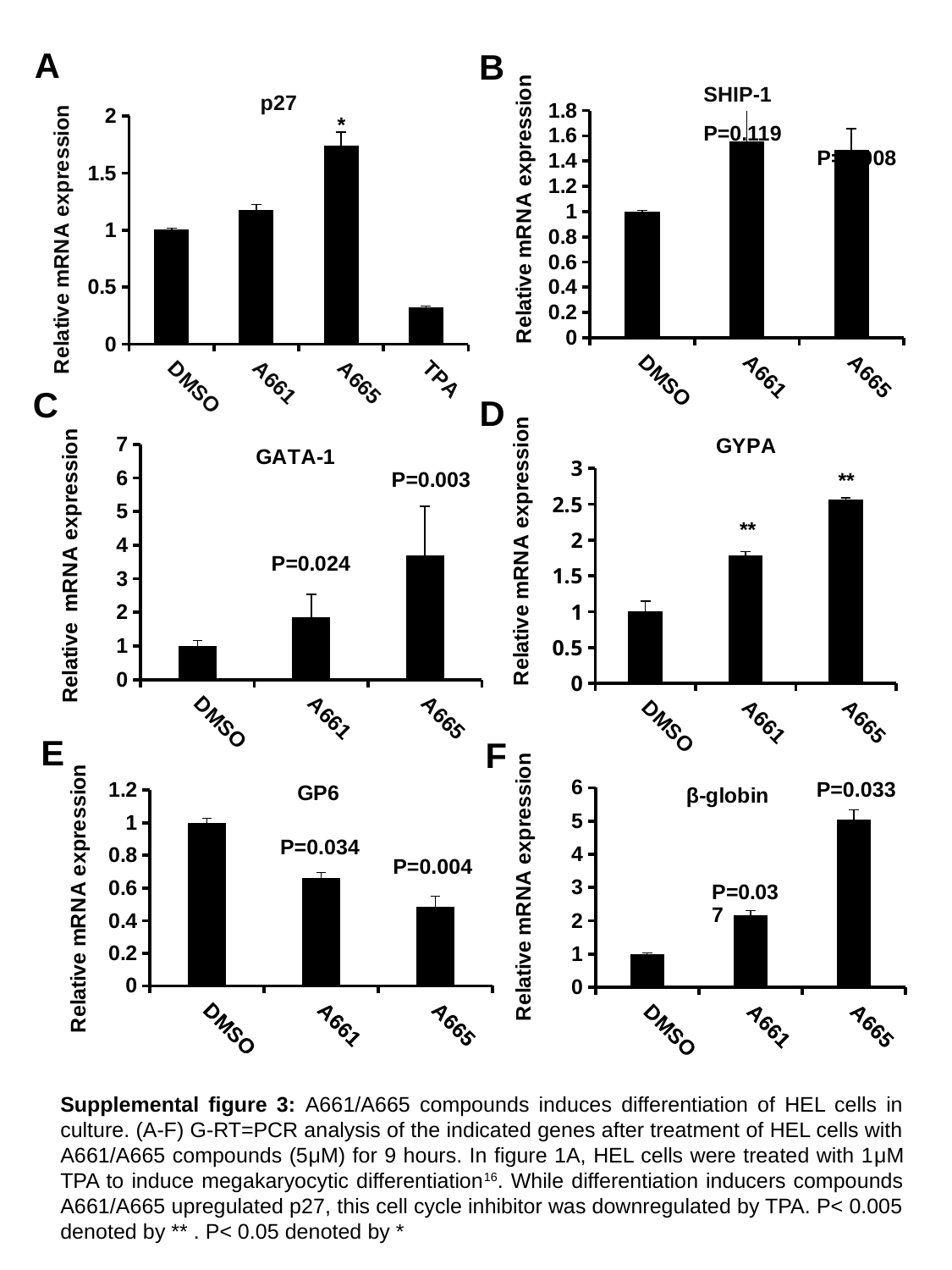

### Chart
| Category | |
|---|---|
| DMSO | 1.0 |
| A661 | 1.5527509350528517 |
| A665 | 1.4853042584440896 |SHIP-1
A
### Chart: p27
| Category | |
|---|---|
| DMSO | 1.000134143012629 |
| A661 | 1.171778453067851 |
| A665 | 1.736537202522723 |
| TPA | 0.316922011446141 |B
*
P=0.119
P=0.008
### Chart
| Category | |
|---|---|
| DMSO | 1.0 |
| A661 | 1.776407202705567 |
| A665 | 2.564212912699769 |C
D
### Chart: GATA-1
| Category | |
|---|---|
| DMSO | 1.0 |
| A661 | 1.8510980505407528 |
| A665 | 3.691935641794649 |
### Chart: β-globin
| Category | |
|---|---|
| DMSO | 1.0 |
| A661 | 2.1532878262378494 |
| A665 | 5.040833046651232 |P=0.033
E
### Chart: GP6
| Category | |
|---|---|
| DMSO | 1.0 |
| A661 | 0.661977961257738 |
| A665 | 0.484321632549351 |F
P=0.034
P=0.004
Supplemental figure 3: A661/A665 compounds induces differentiation of HEL cells in culture. (A-F) G-RT=PCR analysis of the indicated genes after treatment of HEL cells with A661/A665 compounds (5μM) for 9 hours. In figure 1A, HEL cells were treated with 1μM TPA to induce megakaryocytic differentiation16. While differentiation inducers compounds A661/A665 upregulated p27, this cell cycle inhibitor was downregulated by TPA. P< 0.005 denoted by ** . P< 0.05 denoted by *

## Slide 4
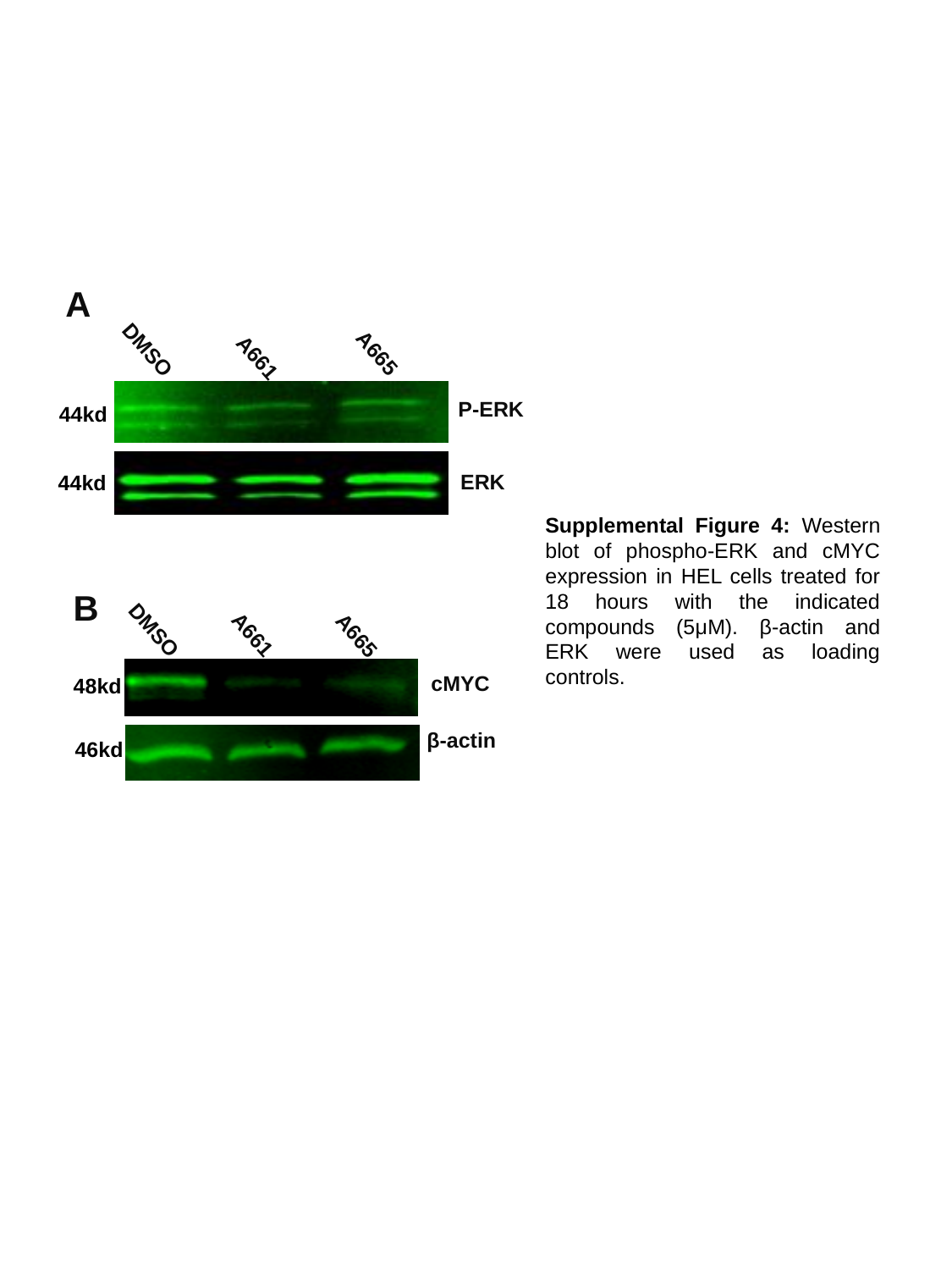

A
DMSO
A665
A661
P-ERK
ERK
44kd
44kd
Supplemental Figure 4: Western blot of phospho-ERK and cMYC expression in HEL cells treated for 18 hours with the indicated compounds (5μM). β-actin and ERK were used as loading controls.
B
DMSO
A661
A665
cMYC
β-actin
46kd
48kd

## Slide 5
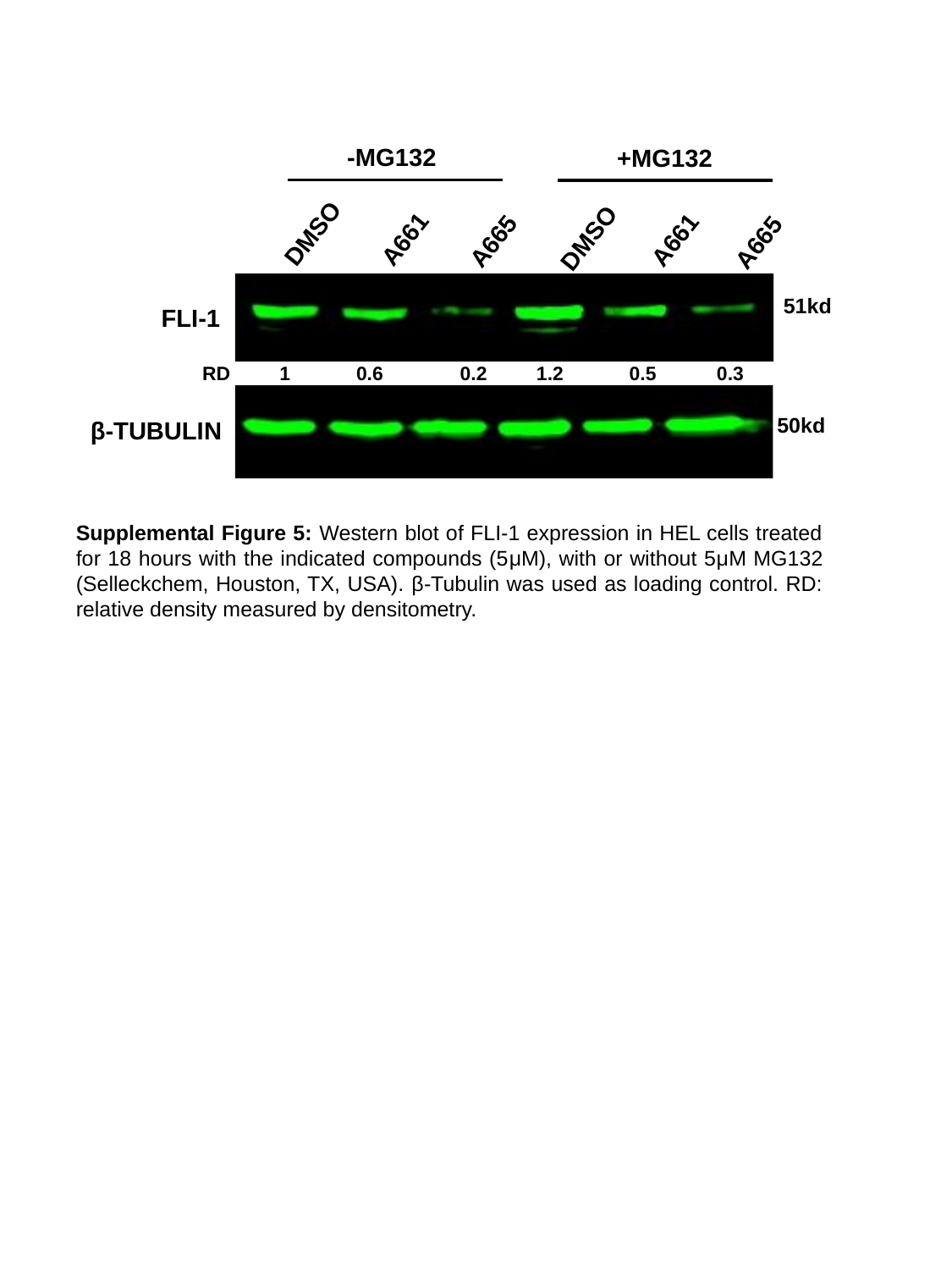

-MG132
+MG132
DMSO
DMSO
A661
A661
A665
A665
51kd
FLI-1
RD 1 0.6 0.2 1.2 0.5 0.3
50kd
β-TUBULIN
Supplemental Figure 5: Western blot of FLI-1 expression in HEL cells treated for 18 hours with the indicated compounds (5μM), with or without 5μM MG132 (Selleckchem, Houston, TX, USA). β-Tubulin was used as loading control. RD: relative density measured by densitometry.

## Slide 6
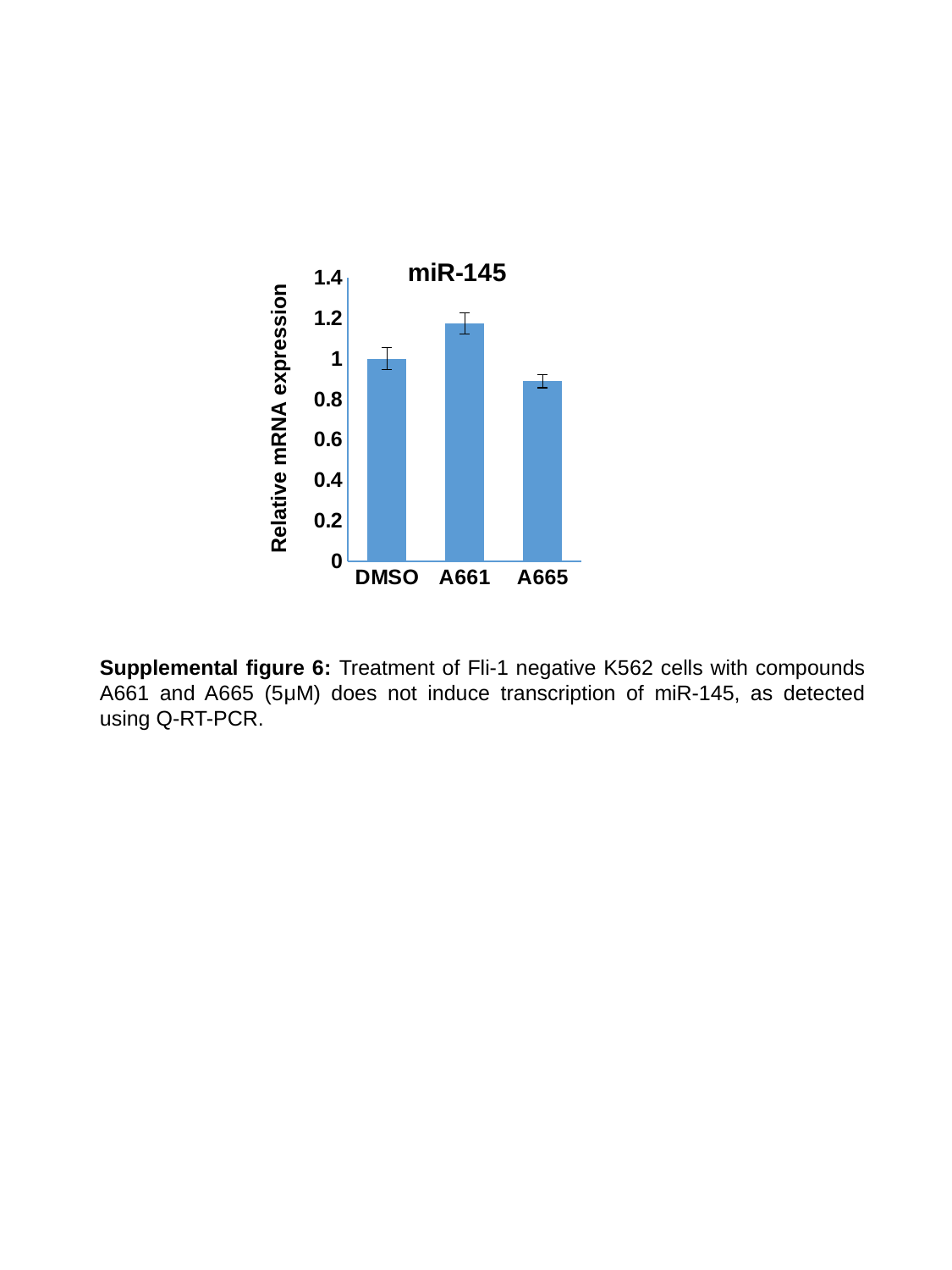

### Chart: miR-145
| Category | MIR145 |
|---|---|
| DMSO | 1.0 |
| A661 | 1.174567588165745 |
| A665 | 0.888797393380506 |Relative mRNA expression
Supplemental figure 6: Treatment of Fli-1 negative K562 cells with compounds A661 and A665 (5μM) does not induce transcription of miR-145, as detected using Q-RT-PCR.
